# Do early‐treated adults with phenylketonuria sense high phenylalanine levels?

**DOI:** 10.1002/jmd2.12446

**Published:** 2024-08-19

**Authors:** Laura Hauri, Raphaela Muri, Regula Everts, Roman Trepp

**Affiliations:** ^1^ Department of Diabetes, Endocrinology, Nutritional Medicine and Metabolism Inselspital, Bern University Hospital and University of Bern Bern Switzerland; ^2^ Division of Neuropediatrics, Development and Rehabilitation, Inselspital Bern Children's University Hospital Bern Switzerland

**Keywords:** cognition, inborn error of metabolism, metabolic control, mood, phenylalanine, phenylketonuria, RCT, sensing

## Abstract

This study aimed to analyze whether early‐treated adults with phenylketonuria (PKU) can subjectively sense high phenylalanine (Phe) concentrations and whether a possible impact of Phe on objective measures of cognitive performance and mood reflects patients' self‐perception. Data from the PICO study, a randomized, placebo‐controlled, double‐blind, crossover trial, were analyzed. Twenty‐eight adults with PKU received either Phe capsules or placebo in two 4‐week intervention periods in a randomized order, with a 4‐week washout in between. The median Phe level increased from 852 μmol/L (interquartile range: 345) to 1455 μmol/L (interquartile range: 369). Neuropsychological assessments were performed at four study visits. At the end of the last study visit, patients were asked whether they could discern the Phe intervention period. Seven of 28 (25%) patients stated that they could not discern between the Phe and the placebo period. Twenty‐one of 28 (75%) patients subjectively thought to sense high Phe levels. Of the 21 patients, 12 (57%) correctly identified the Phe period, whereas 9 (43%) received placebo at the time when they thought they would receive the high Phe load. Binomial tests showed that the probability of 12 out of 21 is *p* = 0.140, and 12 out of 28 is *p* = 0.113. The “Right‐Guess” group showed significantly higher Phe changes than the “Wrong‐Guess” group. Cognitive performance and standardized mood assessment did not significantly differ, and both groups reported similar subjective negative impact on cognition and mood. In conclusion, adults with early‐treated PKU cannot effectively identify high Phe levels, although some individuals may be able to perceive more pronounced increases in Phe levels.

**Synopsis**

The ability of adults with PKU to identify high Phe levels was no better than random.

## 1 INTRODUCTION

Phenylketonuria (PKU) is one of the most common inborn errors of metabolism. Because phenylalanine (Phe) cannot be properly converted to tyrosine, it accumulates in the blood and brain. Left untreated during childhood, this leads to irreversible cognitive and behavioral problems.^1^, ^2^ Nowadays, severe impairments can be prevented by detecting the disease through neonatal screening and following a Phe‐restricted diet during childhood.^3^

Although general agreement exists on the treatment of PKU during pregnancy and childhood, target Phe concentrations during adulthood remain controversial.^4^, ^5^ Several association studies and one small intervention study related high Phe levels to significantly impaired cognition and mood in early‐treated adults with PKU.^6^, ^7^, ^8^ In contrast, an earlier triple‐blinded study in adolescents and our double‐blinded crossover trial in adults, the PICO (Phenylalanine and its Impact on Cognition) study, could not confirm such an impact.^9^, ^10^ The PICO study investigated the effect of high Phe levels on early‐treated adults with PKU in a randomized, placebo‐controlled, double‐blinded design.^5^, ^10^ The 4‐week increase in Phe intake did not significantly affect working memory accuracy, working memory reaction time, manual dexterity, anxiety, vigor, fatigue, anger, and depression scores.^10^ Of note, sustained attention was significantly worsened during high Phe,^10^ and accompanying neuroimaging analyses revealed a reversible decrease of cortical thickness related to the transient high Phe period.^11^ At the same time, functional brain activity was less affected by high Phe levels.^12^

The present substudy aimed to analyze (a) whether patients can subjectively sense elevated Phe levels and (b) whether cognitive performance and mood assessment reflect the patients' self‐perception. We hypothesized that in a double‐blinded study setting, early‐treated adults with PKU cannot distinguish between higher and lower Phe levels.

## 2 METHODS

### 2.1 Patients

This study included data from the PICO study, a randomized, placebo‐controlled, double‐blind, crossover, non‐inferiority trial conducted at the Inselspital, University Hospital Bern, Switzerland.^10^, ^13^ Early‐treated adults with classical PKU were recruited. Participants were excluded from the study if they had not maintained a Phe‐restricted diet for 6 months before the first study visit or had a Phe level above 1600 μmol/L in the preceding 6 months. Other exclusion criteria were pregnancy and treatment with sapropterin or pegvaliase.

### 2.2 Methods

The intervention consisted of two 4‐week oral administrations of Phe and placebo capsules. Eligible patients were randomly allocated (1:1) to either the Phe‐placebo group (4‐week intervention with Phe followed by a 4‐week intervention with placebo) or the placebo‐Phe group (4‐week intervention with placebo followed by a 4‐week intervention with Phe) with a 4‐week washout period between the two interventions (Figure S1).^13^ Patients received capsules containing 250 mg of Phe (or placebo) thrice daily. The total daily amount of Phe (and placebo) was 1500–3000 mg according to sex and weight, intending to mimic the total Phe intake likely to be consumed by the patient being fully «off diet». Four study appointments in weeks 0, 4, 8, and 12 took place before (weeks 0 and 8) and after (weeks 4 and 12) the Phe and placebo intervention periods.

At the first study visit, baseline data (age, sex, IQ) were collected. The IQ was assessed with a short form of the Wechsler Adult Intelligence Scale Fourth Edition (WAIS‐IV), which included the subtests Matrix Reasoning, Vocabulary, Arithmetic, and Symbol Search.^14^ Neuropsychological assessments were conducted at all four study visits, and a blood sample was drawn to measure the Phe concentrations. The evaluation included tasks measuring performance in working memory (n‐back task, Test of Attentional Performance, TAP),^15^ inhibition (time of Stroop task condition 3, D‐KEFS,^16^ and sustained attention (TAP)). In addition, mood was assessed with the Profile of Mood States (POMS) questionnaire.^17^

At the end of the final study visit, patients were asked whether they could discern the Phe from the placebo period. At the end of the last study visit, patients were asked, “Could you discern the Phe from the placebo period?” and if yes: “Did you perceive that the presumed higher Phe levels had negatively impacted your cognitive performance or mood?”

### 2.3 Statistical analysis

Participants who had correctly guessed the Phe period (“Right‐Guess” group) were compared with the ones who incorrectly guessed (“Wrong‐Guess” group). The groups were compared with regard to baseline characteristics (age, sex, IQ, working memory, inhibition, sustained attention, plasma Phe at the first study visit) and changes in cognitive performance (working memory, inhibition, and sustained attention), mood, and plasma Phe. The changes were determined by subtracting the results after the 4‐week high Phe load from the ones before the Phe load.

The binomial test was used to test the null hypothesis that the probability of a correct guess is 0.5 (random chance). After testing the variables for normal distribution with a Shapiro–Wilk test, a two‐tailed Mann–Whitney *U* test or Fisher's exact test was used for the analysis. Effect sizes were interpreted using the rank‐biserial correlation (*r*), where *r* = 0.10 represents a small effect, *r* = 0.30 medium effect, and *r* = 0.50 large effect.^18^ *P*‐values <0.05 were considered statistically significant.

## 3 RESULTS

The demographic and metabolic data of the patients are presented in Table 1. Twenty‐eight patients were included in this study. The median Phe level of the patients increased from 852 μmol/L (interquartile range: 345) to 1455 μmol/L (interquartile range: 369). Twenty‐one patients (75%) claimed to know in which period they received Phe, whereas the remaining 7 (25%) did not. Of the 21 patients who claimed to sense high Phe levels, 12 (57%, 43% of all) were correct (“Right‐Guess”). The remaining 9 (43%, 32% of all) patients guessed the Phe period incorrectly (“Wrong‐Guess”), assuming they were taking Phe while receiving placebo. Binomial tests showed that the probability of exactly 12 out of 21 is *p* = 0.140, and 12 out of 28 is *p* = 0.113. There were no significant differences in age, sex, IQ, baseline Phe levels, or working memory performance between the “Right‐Guess,” the “Wrong‐Guess,” and the “No‐Guess” groups (Table 1).

**TABLE 1:** Baseline and metabolic data.

	Right‐Guess *n* = 12	Wrong‐Guess *n* = 9	No‐Guess *n* = 7
Age (years)	35.1 (7.8)	33.1 (9.1)	31.9 (11.3)
Sex (females)	4 (33%)	5 (66%)	3 (42%)
IQ	96.8 (15.2)	96.4 (9.5)	100 (11.1)
Working memory^a^	93.2 (5.3)	95.8 (4.1)	93.6 (3.9)
Inhibition^b^	51.7 (10.6)	51.8 (9.3)	55.4 (12.0)
Sustained attention^c^	172.5 (53.0)	169.8 (23.2)	181 (43.8)
Plasma Phe (μmol/L)	837.2 (208.1)	760.4 (236.3)	765.7 (295.4)

*Note*: Sex *n* (%), other results mean (SD).

Abbreviations: Phe, phenylalanine; SD, standard deviation.

^a^ Accuracy in %.

^b^ Time in seconds.

^c^ SD of reaction time in milliseconds.

**FIGURE 1:**
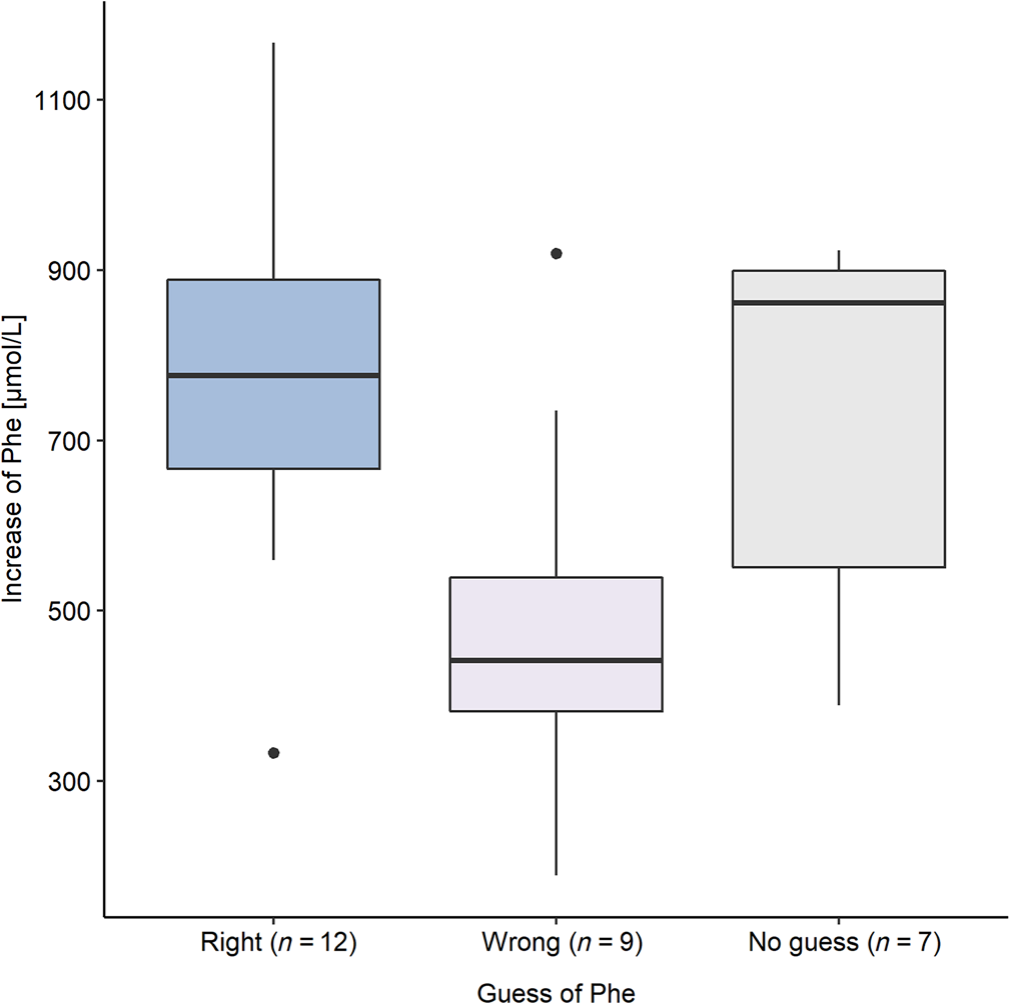
Phenylalanine increase in the “Right‐Guess,” “Wrong‐Guess,” and “No‐Guess” group. Phe, Phenylalanine; boxplots show median, interquartile range, range, and outliers.

The “Right‐Guess” group showed significantly higher Phe levels after the Phe period (*U* = 88.0, *p* = 0.016 *r* = 0.63) and more extensive Phe changes (*U* = 88.0, *p* = 0.016, *r* = 0.63) than the “Wrong‐Guess” group (Figure 1). However, the differences between before and after Phe intake in working memory (*U* = 63.0, *p* = 0.519, *r* = 0.17), inhibition (*U* = 55.5, *p* = 0.914, *r* = 0.03), sustained attention (*U* = 56.5, *p* = 0.859, *r* = 0.05), and mood assessment (*U* = 29, *p* = 0.075, *r* = −0.46), did not differ significantly between the “Right‐Guess” and “Wrong‐Guess” group.

During the phase in which patients anticipated taking Phe, 14 (67%) reported a negative impact on their cognitive performance, and 13 (62%) experienced a negative effect on their mood. Twelve (57%) patients reported that both cognition and mood were affected, 2 (10%) only cognition, and 1 (5%) only mood. The “Right‐Guess” group and the “Wrong‐Guess” group did differ with respect to their report on neither the subjective impact of high Phe on cognition (“Right‐guess group”: 8/12, 66%; “Wrong‐guess” group: 6/9, 66%, *p* = 1) nor mood (“Right‐guess” group: 7/12, 58%; “Wrong‐ guess” group: 6/9, 66%, *p* = 1).

## 4 DISCUSSION

In the present study, 43% of adult patients with PKU could subjectively correctly identify high Phe load over 4 weeks, whereas 57% could not (i.e., either did not feel a difference or guessed wrongly).

The higher Phe concentrations were unrelated to objective or subjective cognitive performance or mood assessment, and the probability that the patients guessed the intervention period correctly was no higher than random guesses. The same question was investigated in an earlier study of adolescents and their parents. In this triple‐blinded study, the answers to the question of whether the Phe values were high or low were also no better than chance.^19^ Of note, in the current study, the “Right‐Guess” group had a significantly higher Phe increase and higher concentrations of Phe after the Phe period. This suggests that patients might be more likely to sense Phe if either Phe levels are very high, Phe changes are high, or both. On the other hand, the “Wrong‐Guess” group reported a subjective negative impact on their cognition and mood to the same degree as the “Right‐Guess” group. This supports the previous notions that the patients' self‐perception of symptoms is a complex issue that depends not only on metabolic control but also on personality and disease acceptance.^20^, ^21^ Expectations are likely to influence the reporting of symptoms. In the PICO study, patients reported significantly more adverse events during Phe administration. However, this was limited to the Phe administration during the first intervention period of the crossover trial—patients who received Phe in the second intervention period showed fewer adverse events than those who received placebo in the first.^10^ The effects of PKU on HRQoL appear to be complex as well. Most early‐treated adults with PKU do not experience a larger impact on health‐related quality of life.^22^ However, patients implement the protein‐restricting diet to varying degrees, entering into a trade‐off between a possible reduction in PKU‐associated symptoms by adhering to the diet and the restrictions this therapy can entail in everyday life.^23^

A limitation of the study is the lack of re‐exposition of the same patients to examine the retest reliability. In particular, it would have been interesting to see to what extent the patients in the “Right‐Guess” group would be correct if they were re‐exposed. Another limitation is that the median Phe level was above 700 μmol/L at baseline and increased into the range of untreated patients during the Phe period. The distinguishability of different Phe levels might differ at different thresholds in adults. In other words, an increase from 300 to 1000 μmol/L could be perceived differently than from 700 to 1400 μmol/L. Further qualitative and quantitative research is needed to investigate whether early‐treated adults with PKU sense Phe differently at varying levels of Phe or with different extents or speeds of changes in Phe levels.

To conclude, adults with early‐treated PKU cannot effectively identify high Phe levels, though some individuals may be able to perceive higher and more pronounced increases in their Phe levels. Even if the possibility of a higher sensitivity cannot be ruled out in individual cases, we advise against suggesting to patients that non‐specific symptoms such as altered cognition or fatigue are likely to be related to current Phe levels.

## AUTHOR CONTRIBUTIONS

LH: performed statistical analysis, wrote article; RM: designed research, conducted research, wrote article; RE: designed research, conducted research, financed research, wrote article, had primary responsibility for final content; RT: designed research, conducted research, finances research, wrote article, had primary responsibility for final content. All authors have read and approved the final manuscript.

## FUNDING INFORMATION

This study was supported by the following foundations: Swiss National Science Foundation (grant 192706 to RE; grant 184453 to RM), Vontobel Foundation (Switzerland, Grant to RT and RE), Bangerter Rhyner Foundation (Switzerland, Grant to RT), Fondation Rolf Gaillard pour la recherche en endocrinologie, diabétologie et métabolisme (Switzerland, Grant to RE), Nutricia Metabolics Research Fund (Netherlands, Grant to RT), and a young investigator grant from the Inselspital Bern (CTU grant). The funder of the study had no role in study design, data collection, data analysis, data interpretation, or writing of the report

## CONFLICT OF INTEREST STATEMENT

None declared.

## ETHICS STATEMENT

Ethical approval was obtained from the local Ethics Committee, Bern, Switzerland. The trial complied with the Declaration of Helsinki and Good Clinical Practice guidelines and was registered with clinicaltrials.gov (NCT03788343).

## Supporting information


**Figure S1.** Design of the PICO study.

## Data Availability

The data that support the findings of this study are available from the corresponding author, [RT], upon reasonable request.
