# Supplementary figures and images for: Do early‐treated adults with phenylketonuria sense high phenylalanine levels?
Source: JIMD Rep. 2024 Aug 19;65(5):354–8. doi: 10.1002/jmd2.12446 (PMC11558469; doi:10.1002/jmd2.12446)

# PICO Study Design

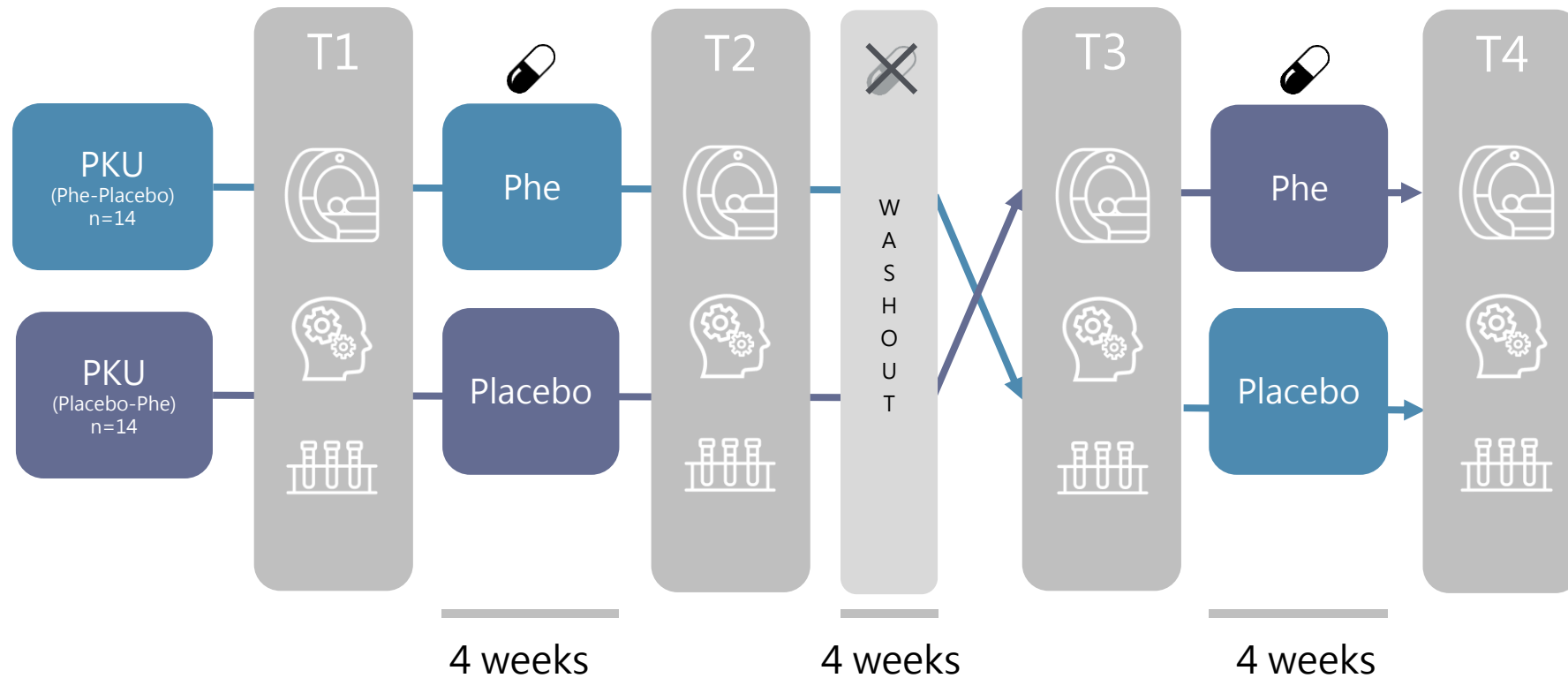

Supplement: Supplementary file 1 — Figure S1. Design of the PICO study. [file JMD2-65-354-s001.pdf]
